# Mitochondrial Dysfunction After Repeated Mild Blast Traumatic Brain Injury Is Attenuated by a Mild Mitochondrial Uncoupling Prodrug

**DOI:** 10.1089/neu.2023.0102

**Published:** 2023-11-09

**Authors:** W. Brad Hubbard, Hemendra J. Vekaria, Gopal V. Velmurugan, Olivia J. Kalimon, Paresh Prajapati, Emily Brown, John G. Geisler, Patrick G. Sullivan

**Affiliations:** ^1^ Lexington Veterans' Affairs Healthcare System, Lexington, Kentucky, USA.; ^2^ Department of Physiology, University of Kentucky, Lexington, Kentucky, USA.; ^3^ Spinal Cord and Brain Injury Research Center, University of Kentucky, Lexington, Kentucky, USA.; ^4^ Department of Neuroscience, University of Kentucky, Lexington, Kentucky, USA.; ^5^ Mitochon Pharmaceuticals, Inc., Blue Bell, Pennsylvania, USA.

**Keywords:** blast injury, dinitrophenol, low-level blast, mitochondria, oxidative stress

## Abstract

Mild traumatic brain injury (mTBI) results in impairment of brain metabolism, which is propagated by mitochondrial dysfunction in the brain. Mitochondrial dysfunction has been identified as a pathobiological therapeutic target to quell cellular dyshomeostasis. Further, therapeutic approaches targeting mitochondrial impairments, such as mild mitochondrial uncoupling, have been shown to alleviate behavioral alterations after TBI. To examine how mild mitochondrial uncoupling modulates acute mitochondrial outcomes in a military-relevant model of mTBI, we utilized repeated blast overpressure of 11 psi peak overpressure to model repeated mild blast traumatic brain injury (rmbTBI) in rats followed by assessment of mitochondrial respiration and mitochondrial-related oxidative damage at 2 days post-rmbTBI. Treatment groups were administered 8 or 80 mg/kg MP201, a prodrug of 2,4 dinitrophenol (DNP) that displays improved pharmacokinetics compared with its metabolized form. Synaptic and glia-enriched mitochondria were isolated using fractionated a mitochondrial magnetic separation technique. There was a consistent physiological response, decreased heart rate, following mbTBI among experimental groups. Although there was a lack of injury effect in mitochondrial respiration of glia-enriched mitochondria, there were impairments in mitochondrial respiration in synaptic mitochondria isolated from the prefrontal cortex (PFC) and the amygdala/entorhinal/piriform cortex (AEP) region. Impairments in synaptic mitochondrial respiration were rescued by oral 80 mg/kg MP201 treatment after rmbTBI, which may be facilitated by increases in complex II and complex IV activity. Mitochondrial oxidative damage in glia-enriched mitochondria was increased in the PFC and hippocampus after rmbTBI. MP201 treatment alleviated elevated glia-enriched mitochondrial oxidative damage following rmbTBI. However, there was a lack of injury-associated differences in oxidative damage in synaptic mitochondria. Overall, our report demonstrates that rmbTBI results in mitochondrial impairment diffusely throughout the brain and mild mitochondrial uncoupling can restore mitochondrial bioenergetics and oxidative balance.

## Introduction

Blast-induced traumatic brain injury (TBI) results in ongoing neurological deficits, including psychiatric and cognitive impairments.^1–3^ Lower levels of blast exposure are relatively common in military settings, including training operations.^4–6^ As there are no current FDA-approved treatments for mild TBI (mTBI), pre-clinical studies have sought to characterize the neuropathobiology underlying mild blast TBI (mbTBI) and extensive focus has been on neurovascular disruption and inflammatory outcomes.^7–13^ Recent efforts have been taken to characterize metabolic and mitochondrial outcomes following mbTBI.^14–16^ However, there is a gap in understanding regarding mitochondrial bioenergetic changes and mitochondrial oxidative damage after bTBI. Further, no study to date has evaluated a therapeutic approach to targeting mitochondrial dysfunction following bTBI.

Several studies have pointed to mitochondrial dysfunction as a key mediator of secondary pathophysiology following bTBI.^15–18^ Deficits in mitochondrial function and metabolism are considered driving forces in progressive energy crisis developed after mTBI.^19–22^ Oxidative stress and mitochondrial dysfunction are intertwined and found to be key players in cellular vulnerability following mTBI.^19,20,23–25^ Indeed, our group has published seminal findings showing that mitochondrial dysfunction in the hippocampus and cortex occurs acutely after mTBI and repeated mTBI leads to overt oxidative damage.^19^ To target both oxidative stress and mitochondrial dysfunction following mTBI, it is critical to understand free radical production and reactive oxygen species (ROS) at the mitochondrial level.

Free radical production is a byproduct of adenosine triphosphate (ATP) generation in mitochondria via the electron transport chain. These free radicals can lead to highly reactive hydroxyl radical (OH^·^) via the Fenton reaction. OH^·^ rapidly attacks unsaturated fatty acids in membranes causing lipid peroxidation and the production of 4-hydroxynonenal (HNE) that conjugates to membrane proteins, impairing their function.^26–29^ In similar pathways, reactive nitrogen intermediates to form 3-nitrotyrosine (3NT). Mitochondrial ROS production is intimately linked to membrane potential (ΔΨ) such that hyperpolarization (high ΔΨ) increases and promotes ROS production.^30–32^ At a high ΔΨ, protons can no longer be pumped out of the matrix (against the electrochemical proton gradient), ultimately resulting in increased ROS production.

ΔΨ is endogenously mediated by uncoupling proteins (UCPs), which function to dissociate ATP production from oxygen consumption,^33^ leading to heat generation. Importantly, mild uncoupling could be beneficial and neuroprotective in neurological disease states because it causes a decrease in ROS production.^30,34–36^ Exogenous mild mitochondrial uncoupling using 2,4 dinitrophenol (DNP), an oral brain-penetrating small molecule, has been shown to be neuroprotective in brain lesion and TBI rodent models.^37,38^ The compound MP201 is a prodrug that in the portal vein gets metabolized to DNP. As a weak acid, DNP is specifically attracted to the pH basic environment of the mitochondria in the cell, thereby releasing its dissociable proton (hydrogen, H^+^) across the mitochondrial membrane into the matrix.^39^ MP201 is a novel prodrug synthesized with an ∼20 × lower C_max_ and ∼3 × longer elimination time compared with the parent drug DNP.^36^ The parent molecule, DNP, is a rapidly absorbed, brain-penetrant small molecule (MW = 184) with wide tissue distribution and likely a Class I molecule not requiring transporters.^36^ Low doses of DNP (MP101, good manufacturing practice (GMP) batch with an open Investigational New Drug (IND) #138612) and MP201 appear to provide broad neuroprotection as seen in positive findings in a host of animal models representing Huntington's disease, Alzheimer's disease, multiple sclerosis, Parkinson's, TBI, and optic neuritis.^39–45^

In our investigation of MP201 to restore brain bioenergetics, there is critical importance of examining mitochondrial sub-populations after TBI, as detailed by our group and others.^46–51^ Overwhelmingly, results have shown that synaptic populations are more vulnerable after TBI as compared with non-synaptic or glia-enriched populations. Indeed, our group has recently shown this in direct comparison from the same mitochondrial samples.^52^ As such, fractionated mitochondrial magnetic respiration (FMMS) will be used to examine synaptic and glia-enriched mitochondria separately in this study.

Based on our previous work showing that two repeated mTBIs can result in brain mitochondrial deficits and oxidative stress,^19^ we use a repeated mild blast TBI (rmbTBI) paradigm with an inter-injury interval of 2 days.^53^ We hypothesize that rmbTBI induces impairment in mitochondrial respiration and mitochondrial-associated oxidative damage in the brain, which can be restored using mild mitochondrial uncoupling. To test this hypothesis, we utilized a model of rmbTBI and conducted a dose-response as well as therapeutic timing study of MP201. We then used FMMS to isolate synaptic and glia-enriched mitochondrial populations to enhance the sensitivity of our assays. Finally, we examined acute mitochondrial respiration and oxidative damage from distinct brain regions, including the prefrontal cortex (PFC), hippocampus, and amygdala/entorhinal cortex/piriform cortex (AEP) region.

## Methods

### Animals and experimental setup

All of the studies performed were approved by the University of Kentucky Institutional Animal Care and Use Committee (IACUC), which is accredited by the Association for the Assessment and Accreditation for Laboratory Animal Care, International (AAALAC, International) and all experiments were performed following its guidelines. All animal experiments were compliant with the Animal Research: Reporting of *In Vivo* Experiments (ARRIVE) guidelines and experiments were carried out in accordance with the National Institutes of Health's Guide for the Care and Use of Laboratory Animals (NIH publication #8023, revised 1978). Male (∼260 g average weight) Sprague-Dawley rats (Charles River) were used at 8 weeks of age.

**FIG. 1.**
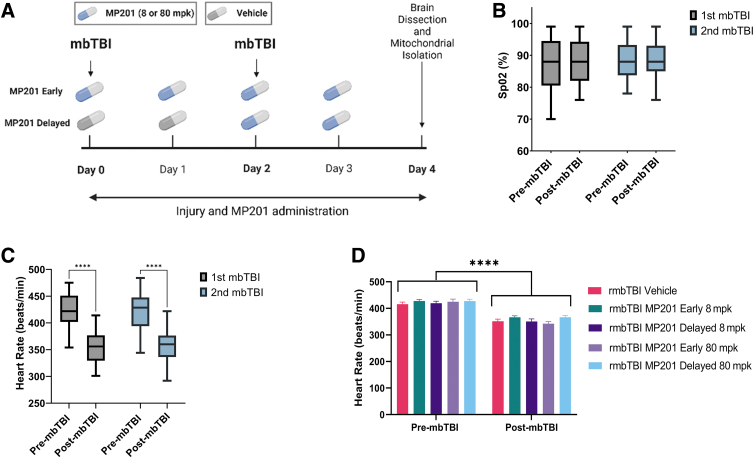
Experimental design and rmbTBI effects on key physiological parameters. **(A)** Experimental design of the study examining the dose-response and therapeutic timing of MP201 treatment after rmbTBI. rmbTBI was induced using two blast exposures at a 2-day inter-injury interval. Assays were conducted at 2 days post-rmbTBI. Created with BioRender.com. **(B)** Physiological recordings were taken 5 min before (pre-mbTBI) and 5 min after (post-mbTBI) mbTBI procedure. Box-and-whisker plot represents the peripheral oxyhemoglobin saturation (SpO_2_) pre-mbTBI and post-mbTBI from male rats after first and second mbTBI (*n* = 40/group; all blast groups combined). **(C)** Box-and-whisker plot represents heart rate pre-mbTBI and post-mbTBI from male rats after first and second mbTBI (*n* = 40/group; all blast groups combined). **(D)** Heart rate pre-mbTBI and post-mbTBI for rmbTBI Vehicle, rmbTBI MP201 Early 8 mpk, rmbTBI MP201 Delayed 8 mpk, rmbTBI MP201 Early 80 mpk, and rmbTBI MP201 Delayed 80 mpk groups (*n* = 8/group). Data are mean ± maximum and minimum (box and whisker) (B, C) or mean ± SEM (D); *p* ≤ 0.001**** by two-way ANOVA with Sidak's post hoc (C, D). ANOVA, analysis of variance; mbTBI, mild blast traumatic brain injury; rmbTBI, repeated mild blast traumatic brain injury; SEM, standard error of the mean.

Animals were randomly assigned to groups, using random number generators (*n* = 8/group). Researchers were blinded to treatment groups during outcome assessment and data analysis. Six experimental groups were included in the study: Sham Vehicle, rmbTBI Vehicle, rmbTBI MP201 Early 8 mpk, rmbTBI MP201 Delayed 8 mpk, rmbTBI MP201 Early 80 mpk, and rmbTBI MP201 Delayed 80 mpk. Early treatment groups received MP201 after the first mbTBI procedure and then daily, whereas delayed treatment groups received vehicle after the first mbTBI procedure and then MP201 after the second mbTBI procedure daily (Fig. 1). Experiments were performed in four distinct cohorts of 12 animals each. Each cohort contained *n* = 2 from all six experiment groups. The animals were housed two per cage and maintained in a 12 h light/12 h dark cycle. Confounding factors were minimized by including various treatment groups in the same cage, ensuring all experimental groups were operated on/analyzed at the same time (especially if the assay required multiple cohorts of animals), and all animals were housed in the same room. All animals were fed a balanced diet *ad libitum* and water was reverse osmosis generated. The exact number of animals per study is reported within the figure legends. For additional details on common data elements used, see the publication by Hubbard and colleagues.^12^ For all assays, technical replicates were included.

### Repeated mild blast TBI model

Methods were based on model optimization as detailed by Hubbard and colleagues.^12^ The McMillan Blast Device (MBD) was used to generate the blast overpressure wave using compressed helium and active rupture of Mylar sheets. Pressure parameters (Table 1) were based on recordings from a pitot probe (face-on/reflected and side-on) pressure (custom built, Stumptown Research & Development, LLC, Black Mountain, NC, USA; XTL-190S-100A, Kulite Semiconductor Products, Inc., Leonia, NJ, USA) and piezo-resistive side-on sensors (model #XTEL-100-190S-100A; Kulite). Data from each sensor were routed directly to the TMX-18 (AstroNova, Inc., West Warwick, RI, USA) and analyzed using AstroView software (AstroNova, Inc.).

**Table 1.** Parameters of Blast Exposure Based on Experimental Group

Experimental group	Blast day	Peak static overpressure (psi)	Positive overpressure duration (msec)	Positive impulse (psi^*^msec)	Peak total overpressure (psi)	Peak dynamic overpressure (psi)
Blast Vehicle	1st Blast	11.81 ± 1.29	6.63 ± 0.32	37.40 ± 3.47	24.02 ± 4.44	12.22 ± 3.21
	2nd Blast	11.99 ± 0.67	6.44 ± 0.16	37.49 ± 1.64	24.28 ± 1.70	12.29 ± 1.23
	
Early 8 mpk	1st Blast	11.56 ± 1.38	6.59 ± 0.20	36.45 ± 3.06	23.17 ± 3.24	11.61 ± 1.98
	2nd Blast	10.99 ± 1.33	6.51 ± 0.26	35.15 ± 2.38	21.62 ± 3.52	10.63 ± 2.25
	
Delayed 8 mpk	1st Blast	11.98 ± 0.64	6.50 ± 0.23	38.21 ± 1.41	24.87 ± 2.15	12.89 ± 1.41
	2nd Blast	10.45 ± 2.43	6.74 ± 0.43	35.90 ± 3.04	21.58 ± 3.99	11.13 ± 1.69
	
Early 80 mpk	1st Blast	11.45 ± 1.18	6.64 ± 0.33	38.16 ± 1.86	22.72 ± 3.73	11.26 ± 2.59
	2nd Blast	11.03 ± 0.84	6.50 ± 0.25	36.17 ± 2.06	21.58 ± 2.70	10.55 ± 1.89
		
Delayed 80 mpk	1st Blast	11.03 ± 1.95	6.69 ± 0.38	36.58 ± 3.32	21.48 ± 5.80	10.44 ± 4.00
	2nd Blast	11.83 ± 0.36	6.46 ± 0.13	37.04 ± 1.85	23.52 ± 2.36	11.68 ± 2.20

Sprague-Dawley rats were transported and temporarily housed in a climate-controlled room enclosed away from the blast tests and had access to food and water *ad libitum* throughout the course of the experiment. Immediately prior to injury, the rats were anesthetized with isoflurane using a SomnoSuite Low-Flow Anesthesia System (Kent Scientific Corp., Torrington, CT, USA). Rats received 900 mL/min flow at 4.0% isoflurane until fully anesthetized and then were placed on a nose cone (400 mL/min at 2.5% isoflurane) for physiological recordings. Rats were placed into a mesh netting support (Industrial Netting, Minneapolis, MN, USA) and secured into the MBD (2 ft from open end of tube) laterally with the left side facing the blast.^54^ Once loaded into the MBD, the rat's body was protected from direct forces by a steel tube that surrounded the body but left the head completely exposed to the blast. The rats were subjected to compressed helium-driven blasts at 11 psi peak static overpressure (blast parameters for each group are detailed in Table 1) to model mbTBI. Physiological recordings were taken using SomnoSuite technology (MouseSTAT) 5 min before and 5 min after the mbTBI procedure. Sham animals had all procedures performed, except for blast exposure. Rats were monitored and recovered before return transportation.

### MP201 administration

MP201 was formulated at either 8 or 80 mg/kg in the vehicle solution (mixture of 99% 0.5% methyl cellulose and 1% dimethyl sulfoxide [DMSO; CHEBI:28262]).^38^ Rats were given 10 mL/kg or approximately 2.5 mL of solution (depending on weight) at each dose of MP201, or vehicle depending upon group designation. MP201 was administered by oral gavage according to the experimental design (Fig. 1). MP201 was given 15 min post-mbTBI on the days of blast exposure.

### Fractionated mitochondrial magnetic separation

FMMS was completed to fractionate synaptic and glia-enriched mitochondria based on recent publications^50–52^ with key modifications for rat brain tissue. Unilateral hippocampus, PFC, and AEP regions were dissected following CO_2_ exposure. Tissues were homogenized and centrifuged at 1300*g* for 3 min. This homogenate contains free-floating glia-enriched as well as intact synaptosomes. The supernatant was placed in a fresh tube and the pellet was resuspended in isolation buffer to be spun again at 1300*g* for 3 min. The supernatants from the first and second spins were incubated with anti-Tom22 microbeads (Miltenyi Biotec) at a concentration of 8 μL for every 1 mg of starting wet-tissue weight for 30 min. Brain tissue of 8 μL/mg was determined as the optimal concentration that ensured all free mitochondria were captured.

The mixture was then added to MACS separation LS columns, attached to a Quadro MACS Separator (catalog #130–097-040, Miltenyi Biotec) to capture free mitochondria (glia-enriched fraction). The column was plunged and the resulting sample was transferred to a 1.5 μL microcentrifuge tube and centrifuged at 13 ,000*g* for 10 min at 4°C. Meanwhile, the eluate (containing synaptosomes) from the LS columns was spun at 13, 000*g* for 10 min before being placed into the pressurized nitrogen cell disruptor. Once the synaptosomes burst, the resulting solution was incubated with anti-Tom22 microbeads (3 μL for every 1 mg of starting tissue) for 30 min. The mixture was then added to new MACS separation LS columns, attached to a Quadro MACS Separator, plunged, and spun down for protein quantification.

### Mitochondrial respirometry using Seahorse XFe96

Mitochondrial bioenergetics were assayed in isolated mitochondria according to previous studies.^51,52^ Briefly, Seahorse XFe96 Flux Analyzer (Agilent Technologies, Santa Clara, CA, USA) was used to determine oxygen consumption rates (OCRs) in the presence of mitochondrial substrates, inhibitors, and uncouplers. On the day before the assay, the sensor cartridge was hydrated. On the day of the assay, injection ports A to D of the sensor cartridge were loaded to measure the OCR in various mitochondrial respiration states. Chemical stocks were diluted appropriately in respiration buffer (RB; 125 mM KCl, 0.1% bovine serum albumin [BSA], 20 mM HEPES, 2 mM MgCl_2_, and 2.5 mM KH_2_PO_4_, adjusted pH 7.2) to make the final concentration of the chemicals 5 mM pyruvate, 2.5 mM malate, and 2 mM adenosine diphosphate (ADP; via Port A; State III_C1_), 2.5 μM oligomycin A (via Port B; State IV), 4 μM carbonyl cyanide-p-trifluoromethoxyphenylhydrazone (FCCP; via Port C; State V_C1_), and 1 μM rotenone and 10 mM of succinate (via Port D; State V_CII_).

Next, 3 μg non-synaptic and 6 μg synaptic mitochondrial protein, as measured using a bicinchoninic acid (BCA) protein assay (#23227, Pierce), were loaded per well in a volume of 30 μL. Plates were centrifuged and RB was gently added for a total volume of 175 μL in each Seahorse XFe96 well. OCRs were measured based on additions in each injection port. Raw OCR values were used for analysis within a given experiment and reported in all figures. Plate-to-plate and day-to-day variation may result in variable absolute OCR values between experiments, which necessitated the use of blocking factor in statistical analysis.

### Oxidative damage measurements

Mitochondrial homogenate aliquots (unused during respiration assays) were stored at −20°C until utilization for oxidative stress dot-blots. In these samples, 3-nitrotyrosine (3NT) and 4-hydroxynonenal (HNE) were assessed as previously described.^19,52^ Protein concentrations were determined using a BCA protein assay kit (catalog #23227, Thermofisher). Dot-blots were made from 500 ng of mitochondrial lysate per sample in a 96-well Bio-Dot microfiltration apparatus (Bio-Rad). Nitrotyrosine polyclonal antibody (1:2500; catalog #A-21285, Thermofisher) and anti-4 HNE antiserum (1:2500; catalog #HNE11-S, Alpha Diagnostic International, San Antonio, TX, USA) were used along with IRDye 800 CW goat anti-rabbit IgG (1: 10,000; catalog #926-3221, LI-COR Biosciences, USA) secondary antibody to detect 4-HNE and 3-NT expressions from mitochondrial lysates. The membranes were scanned and dot-blot intensities were quantified using a LI-COR DLx Odyssey imaging system.

### Mitochondrial complex activity quantification

Methods were adapted from those of Kalimon and colleagues.^55^ Frozen mitochondrial aliquots were utilized for the assessment of mitochondrial electron transport chain complexes in the Seahorse XFe96 Flux Analyzer. Mitochondria were thawed on ice prior to dilution in Mir05 buffer (0.5 mM EGTA, 3 mM MgCl_2_, 60 mM lactobionic acid, 20 mM taurine, 10 mM KH_2_PO_4_, 20 mM HEPES, 110 mM sucrose). Mitochondria (1.5 μg) were resuspended in 75 μL of Mir05 buffer and spun at 3000*g* for 10 min. The OCR measurements were carried out using Seahorse XFe96 after topping up the wells with 100 μL of MiR05 to final concentrations of alamethicin (35 μg/mL), nicotinamide adenine dinucleotide (NADH; 3.5 mM), and cytochrome c (17.52 μM). Remaining substrates and inhibitors of the electron transport chain were prepared in RB without BSA and loaded into the injection ports as followed: (A) rotenone (0.8 μM) and succinate (10 mM), (B) antimycin A (1 μM), (C) ascorbate (20 mM) and N,N,N′,N′-tetramethyl-p-phenylenediamine (TMPD; 5 mM), and (D) sodium azide (549.3 mM). Complex I activity was derived from the NADH reading minus the antimycin A reading. Complex II activity was calculated from the succinate reading minus the antimycin A reading. Complex IV activity was derived from the ascorbate/TMPD reading minus the sodium azide reading.

### Statistical analysis

Power analysis was conducted (using G*Power statistical software, version 3.0.10) for all experimental data and was based on previous published literature from our group. Analysis was completed based on the analysis of variance (ANOVA) statistical tests and output of F score. *A priori* analysis was performed and effect size was calculated based on expected mean ± standard deviation (SD) within each group. Sample size was calculated using the following parameters: α = 0.05, 1 − β = 0.8, and SD 20% of mean for experimental groups. Primary outcomes for sample size determination were State III mitochondrial bioenergetics. Statistical analysis was performed using Graph Pad Prism (GraphPad Software, CA, USA) or JMP 12 (SAS, NC, USA). For all analyses, a significant difference among groups was defined as *p* < 0.05. For each measure, data were measured using interval/ratio scales. The Brown-Forsythe and Bartlett's tests were performed to ensure homogeneity of variance. Further, the Shapiro-Wilk test was completed to ensure normality. As these criteria were met for all experimental data, parametric statistics were employed for all analyses. Two-way ANOVA with Sidak's post hoc or one-way ANOVA test with Dunnett's post hoc were employed, where appropriate.

## Results

There were no significant differences in peak static overpressure between treatment groups (Table 1). To assess the effect of rmbTBI on acute physiology, oxygen saturation and heart rate monitoring were assessed before and following mbTBI (Fig. 1). There were no decreases in SpO_2_ between pre- and post-blast for either first or second mbTBI. Similar decreases in heart rate after mbTBI were observed after the first and second mbTBI procedures. Also, there was a main effect of time (pre- vs. post-blast) in the heart rate analysis that was not significantly different between treatment groups, indicating similar physiological responses to blast exposure.

**FIG. 2.**
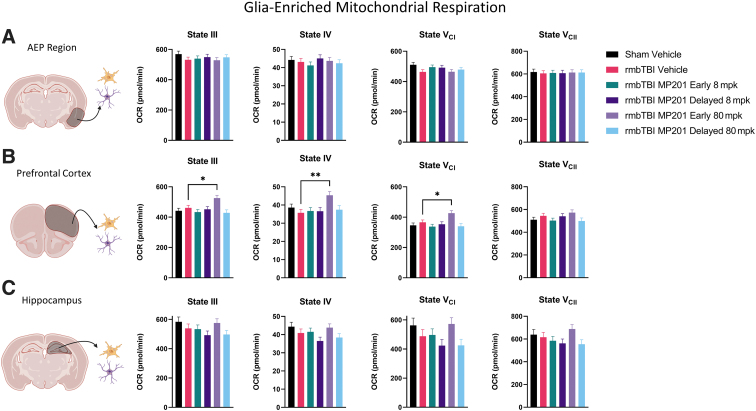
rmbTBI does not result in impaired glia-enriched mitochondrial respiration from various brain regions. Seahorse XFe96 was utilized to measure distinct states of mitochondrial respiration in glia-enriched mitochondria at 2 days following rmbTBI and subsequent MP201 treatment. **(A)** State III, State IV, State V_CI_, and State V_CII_ respiration in glia-enriched mitochondria from the AEP region (*n* = 7–8/group). **(B)** State III, State IV, State V_CI_, and State V_CII_ respiration in glia-enriched mitochondria from the PFC (*n* = 7 − 8/group). **(C)** State III, State IV, State V_CI_, and State V_CII_ respiration in glia-enriched mitochondria from the hippocampus (*n* = 7–8/group). Data represented as mean ± SEM; *p* ≤ 0.05*, *p* ≤ 0.01** by one-way ANOVA with Dunnett's post hoc compared with rmbTBI Vehicle (B). AEP, amygdala/entorhinal/piriform cortex; ANOVA, analysis of variance; OCR, oxygen consumption rate; PFC, prefrontal cortex; rmbTBI, repeated mild blast traumatic brain injury; SEM, standard error of the mean.

Mitochondrial respiration was examined in glia-enriched mitochondria from the AEP region, PFC, and the hippocampus at 2 days post-rmbTBI (Fig. 2). There were no significant differences in glia-enriched mitochondrial function between experimental groups for the AEP region or the hippocampus. Although there was no injury effect (Sham Vehicle vs. rmbTBI Vehicle) in the PFC, rmbTBI MP201 Early 80 mpk had elevated OCR levels as compared with rmbTBI Vehicle for States III, IV, and V_CI_. This provides evidence that early 80 mpk MP201 dosing can elevate mitochondrial function in glia-enriched mitochondria.

**FIG. 3.**
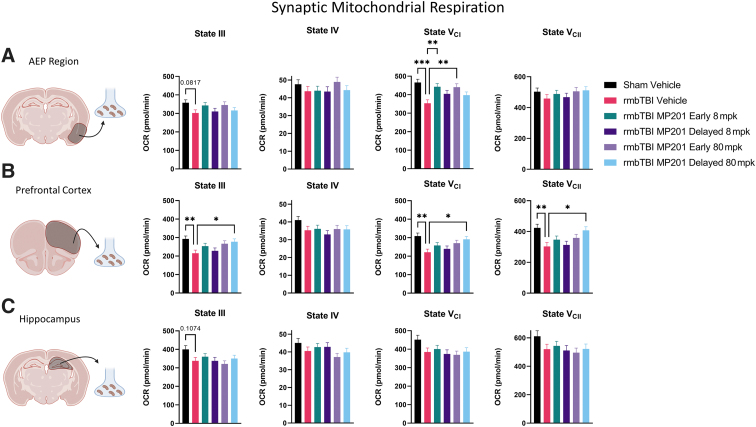
rmbTBI results in robust deficits in synaptic mitochondrial bioenergetics that are rescued by MP201 treatment. Seahorse XFe96 was utilized to measure distinct states of mitochondrial respiration in synaptic mitochondria at 2 days following rmbTBI and subsequent MP201 treatment. **(A)** State III, State IV, State V_CI_, and State V_CII_ respiration in synaptic mitochondria from the AEP region (*n* = 7–8/group). **(B)** State III, State IV, State V_CI_, and State V_CII_ respiration in synaptic mitochondria from PFC (*n* = 7–8/group). **(C)** State III, State IV, State V_CI_, and State V_CII_ respiration in synaptic mitochondria from the hippocampus (*n* = 7–8/group). Data represented as mean ± SEM; *p* ≤ 0.05*, *p* ≤ 0.01**, *p* ≤ 0.001*** by one-way ANOVA with Dunnett's post hoc compared with rmbTBI Vehicle (A, B). AEP, amygdala/entorhinal/piriform cortex; ANOVA, analysis of variance; OCR, oxygen consumption rate; PFC, prefrontal cortex; rmbTBI, repeated mild blast traumatic brain injury; SEM, standard error of the mean.

Growing evidence demonstrates that there are overt deficits in mitochondrial function in neuronal synapse after TBI. To this end, mitochondrial respiration was examined in synaptic mitochondria from the AEP region, PFC, and hippocampus (Fig. 3). There was a marked decrease in State V_CI_ in rmbTBI Vehicle as compared with Sham Vehicle in synaptic mitochondria from the AEP region. rmbTBI MP201 Early 8 mpk and rmbTBI MP201 Early 80 mpk displayed significantly increased State V_CI_ levels as compared with rmbTBI Vehicle in the AEP region. In the PFC, rmbTBI Vehicle resulted in significant decreases in synaptic bioenergetics for States III, V_CI_, and V_CII_ as compared with Sham Vehicle. rmbTBI MP201 Delayed 80 mpk significantly increased mitochondrial function in States III, V_CI_, and V_CII_ as compared with rmbTBI Vehicle. There was no effect of injury (Sham Vehicle vs. rmbTBI Vehicle) in synaptic mitochondrial respiration in the hippocampus. These data show that rmbTBI results in synaptic mitochondrial dysfunction in cortex and amygdala regions. Further, early or delayed MP201 treatment can restore mitochondrial function to sham levels in a region-specific manner.

**FIG. 4.**
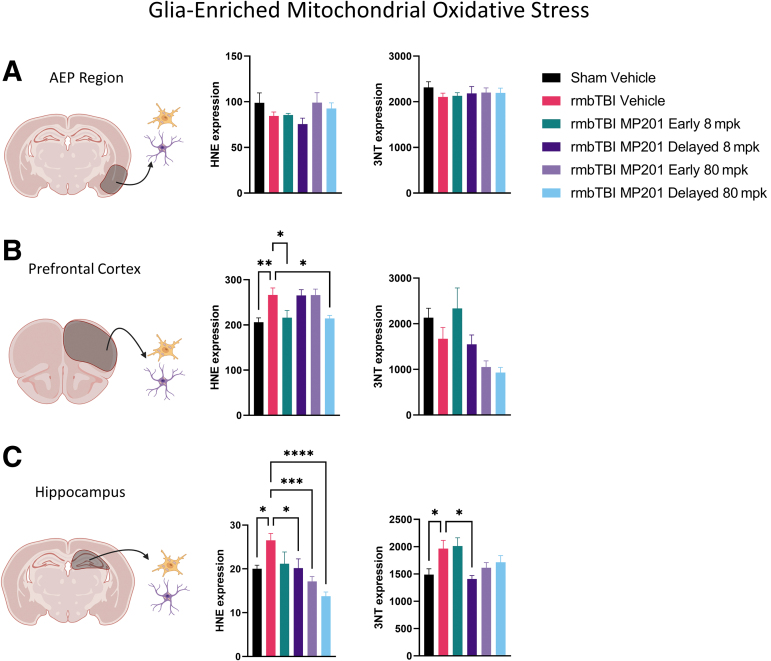
rmbTBI results in elevated levels of mitochondrial oxidative damage in glia-enriched mitochondria that are diminished with MP201 treatment. Dot-blots were performed on glia-enriched mitochondria to measure HNE and 3NT at 2 days following rmbTBI and subsequent MP201 treatment. **(A)** HNE and 3NT levels in glia-enriched mitochondria from the AEP region (*n* = 7–8/group). **(B)** HNE and 3NT levels in glia-enriched mitochondria from the PFC (*n* = 7–8/group). **(C)** HNE and 3NT levels in glia-enriched mitochondria from the hippocampus (*n* = 7–8/group). Data represented as mean ± SEM; *p* ≤ 0.05*, *p* ≤ 0.01**, *p* ≤ 0.001***, *p* ≤ 0.0001**** by one-way ANOVA with Dunnett's post hoc compared with rmbTBI Vehicle (B,C). 3NT, 3-nitrotyrosine; AEP, amygdala/entorhinal/piriform cortex; ANOVA, analysis of variance; HNE, 4-hydroxy-2-nonenal; PFC, prefrontal cortex; rmbTBI, repeated mild blast traumatic brain injury; SEM, standard error of the mean.

One other key hallmark of mbTBI pathophysiology is oxidative damage in the brain, and mitochondria are key epicenters that maintain oxidative balance. Oxidative stress markers, 3NT and HNE, were measured in glia-enriched mitochondrial aliquots (Fig. 4). There were no rmbTBI-induced differences for either 3NT or HNE in the AEP region. In the PFC, there were significantly increased levels of HNE in glia-enriched mitochondria from the rmbTBI Vehicle group as compared with Sham Vehicle. Both rmbTBI MP201 Early 8 mpk and rmbTBI MP201 Delayed 80 mpk lowered HNE levels in PFC glia-enriched mitochondria as compared with rmbTBI Vehicle. In the hippocampus, there was significantly increased levels of both HNE and 3NT in glia-enriched mitochondria from the rmbTBI Vehicle group as compared with Sham Vehicle. rmbTBI MP201 Delayed 8 mpk lowered HNE and 3NT levels in hippocampal glia-enriched mitochondria as compared with rmbTBI Vehicle. Further, rmbTBI MP201 Early 80 mpk and rmbTBI MP201 Delayed 80 mpk significantly lowered HNE levels in hippocampal glia-enriched mitochondria as compared with rmbTBI Vehicle. Overall, these results demonstrated overt increases in oxidative damage in glia-enriched mitochondria following rmbTBI, and MP201 treatment can target and diminish this oxidative damage.

**FIG. 5.**
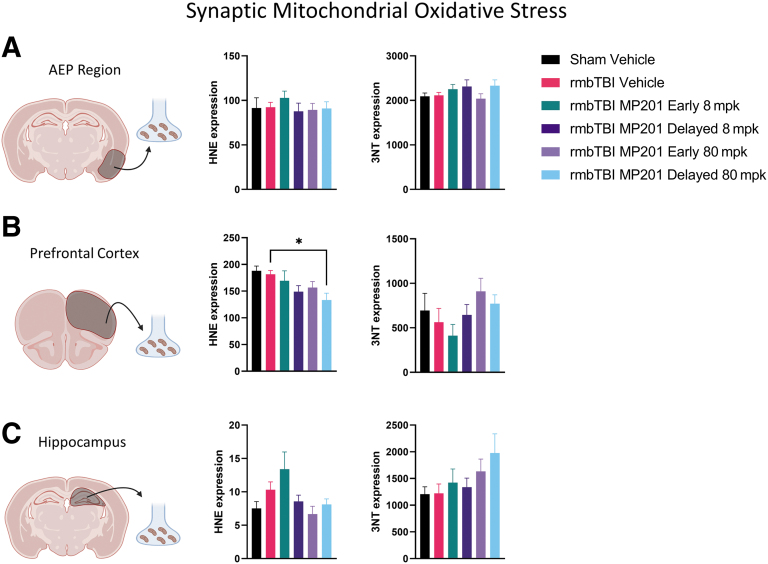
rmbTBI does not alter levels of mitochondrial oxidative damage in synaptic mitochondria from various brain regions. Dot-blots were performed on synaptic mitochondria to measure HNE and 3NT at 2 days following rmbTBI and subsequent MP201 treatment. **(A)** HNE and 3NT levels in synaptic mitochondria from the AEP region (*n* = 7–8/group). **(B)** HNE and 3NT levels in synaptic mitochondria from the PFC (*n* = 7–8/group). **(C)** HNE and 3NT levels in synaptic mitochondria from the hippocampus (*n* = 7–8/group). Data represented as mean ± SEM; *p* ≤ 0.05* by one-way ANOVA with Dunnett's post hoc compared with rmbTBI Vehicle (B). 3NT, 3-nitrotyrosine; AEP, amygdala/entorhinal/piriform cortex; ANOVA, analysis of variance; HNE, 4-hydroxy-2-nonenal; PFC, prefrontal cortex; rmbTBI, repeated mild blast traumatic brain injury; SEM, standard error of the mean.

Further, oxidative damage markers, 3NT and HNE, were assessed in synaptic mitochondrial aliquots (Fig. 5). There were no alterations in either 3NT or HNE following rmbTBI in the AEP or hippocampal regions. There was also no change in 3NT for the PFC; however, rmbTBI MP210 Delayed 80 mpk resulted in significantly lower synaptic HNE levels as compared with rmbTBI Vehicle. Overall, our results indicate that rmbTBI does not alter oxidative damage in synaptic mitochondria. Additionally, 80 mpk MP201 can lower HNE levels in synaptic mitochondria.

**FIG. 6.**
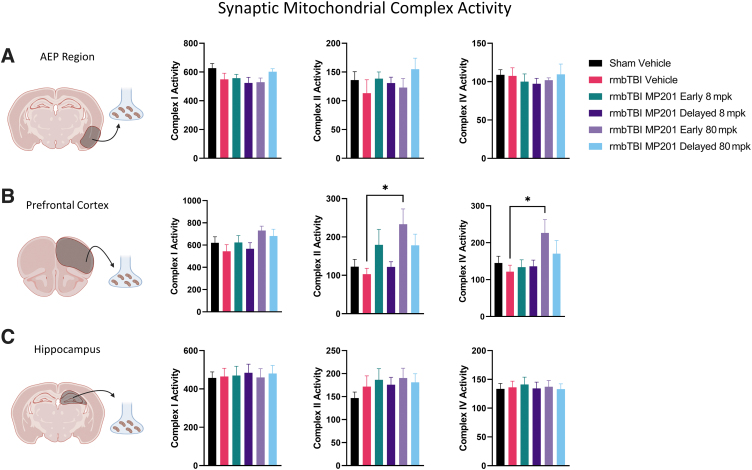
MP201 produces increased mitochondrial complex II and complex IV activity in the PFC despite lack of rmbTBI-induced deficits. Mitochondrial complex activity was measured on frozen mitochondrial aliquots in Seahorse XFe96. Samples were obtained 2 days following rmbTBI and subsequent MP201 treatment. **(A)** Complex I, complex II, and complex IV activity levels in synaptic mitochondria from the AEP region (*n* = 6–8/group). **(B)** Complex I, complex II, and complex IV activity levels in synaptic mitochondria from the PFC (*n* = 6–8/group). **(C)** Complex I, complex II, and complex IV activity levels in synaptic mitochondria from the hippocampus (*n* = 6–8/group). Data represented as mean ± SEM; *p* ≤ 0.05* by one-way ANOVA with Dunnett's post hoc compared with rmbTBI Vehicle (B). AEP, amygdala/entorhinal/piriform cortex; ANOVA, analysis of variance; PFC, prefrontal cortex; rmbTBI, repeated mild blast traumatic brain injury; SEM, standard error of the mean.

To examine whether alterations in synaptic mitochondrial bioenergetics are due to changes in mitochondrial complex activity, we employed a functional assay to examine mitochondrial complex activity from mitochondrial pellets (Fig. 6). There were no alterations in either complex I, II, or IV activity in the AEP or hippocampal regions. Although there were no rmbTBI-induced alterations in complex activity in the PFC, rmbTBI MP201 Early 80 mpk significantly increased complex II and IV activity as compared with rmbTBI Vehicle. These data indicate that rmbTBI does not alter synaptic mitochondrial complex activity but 80 mpk MP201 can increase activity levels in complexes II and IV in synaptic mitochondria.

## Discussion

Physiological changes following multiple mbTBIs are similar to those observed following a single mbTBI,^12^ demonstrating a consistent physiological response that is not exacerbated or mitigated by previous blast exposure. As described in Reneer and associates,^54^ helium displacement of air during blast exposure can create a brief (1 msec up to 1–2 sec) hypoxic environment, but this does not translate into any meaningful physiological outcomes for the animal as oxygen saturation was not altered after either the first or second mbTBI. These results demonstrate the mild severity of blast exposure used in this study.

Past research has identified mitochondrial dysfunction as a key determinant of cellular vulnerability to repeated mTBIs.^19,20^ Our research found that when a second mTBI is sustained during the period of decreased mitochondrial function, oxidative damage is propagated in these vulnerable regions of the brain.^19^ In our study, we found that rmbTBI results in acute synaptic mitochondrial dysfunction and acute glia-enriched mitochondrial oxidative stress. These results confirm that mitochondrial impairment is a key hallmark of pre-clinical mTBI models, including mbTBI.

Other studies have found metabolic crisis, such as altered ATP and decreased cerebral metabolic rate of glucose utilization (CMRglu) after blast exposure in rodents and humans, respectively.^14,56^ Importantly, dysregulation of mitochondrial genes has been reported following blast exposure in military personnel.^57^ A recent study has described long-term changes in pathway enrichment of oxidative phosphorylation and mitochondrial dysfunction in the amygdala following repeated blast exposure.^17^ Together, these studies point to a pivotal role of mitochondria in the long-term pathophysiology and cognitive impairment after mild blast-induced neurotrauma and mild impact-induced TBI.

Many pre-clinical studies targeting mitochondrial dysfunction after TBI have proven successful, including pharmacological modulation of mitochondrial physiology by uncoupling agents, such as MP201 and DNP. In brief, extrinsic mitochondrial uncoupling agents can increase proton leakage across the mitochondrial inner membrane, thereby lowering ΔΨm and reducing oxidative stress.^58–60^ Our group has published reports outlining neuroprotective therapy of the mitochondrial uncouplers FCCP and DNP.^37,61–63^ Mechanistically, treatment with MP201, as a prodrug of DNP, produces reduction in oxidative damage, mitigates mitochondrial Ca^2+^ overloading, and increased mitochondrial respiration, while lowering the mechanistic target of rapamycin (mTOR) to promote mitophagy.^64^ These molecules, while lowering damage, also promote repair by induction of cyclic adenosine monophosphate (cAMP), cAMP-response element binding protein (CREB), and brain-derived neurotrophic factor (BDNF).^40,64,65^ Collectively, this pharmacology appears pan-neuroprotective at low doses and may be neuroprotective with aging.^34,66^

In the current study, we found rampant improvements in both mitochondrial function and oxidative balance after MP201 administration. MP201 was effective at treating injury-related deficits in glia-enriched oxidative stress and synaptic mitochondrial bioenergetics. Strikingly, MP201 was effective in its improvements regardless of whether there were injury-induced deficits. MP201 increased glia-enriched mitochondrial bioenergetics, decreased synaptic HNE expression, and increased synaptic complex II and IV activity, in spite of a lack of injury impairments. Overall, mild mitochondrial uncoupling is an efficacious strategy at targeting acute mitochondrial impairments following mbTBI, which can facilitate cellular recovery.

It is of critical importance to understand the therapeutic window of opportunity following TBI.^51,67^ In the current study, we sought to understand whether mitochondrial dysfunction needed to be targeting at the first mbTBI (early treatment) and resultant metabolic cascade or if treatment following the second mbTBI was sufficient (delayed treatment). Overall, both early and delayed treatment improved outcome measures after rmbTBI, although these increases were brain-region dependent. Further, dose-response studies are key to understanding therapeutic targets.^68,69^ We found that both 8 and 80 mg/kg MP201 were effective at alleviating dysfunction, but 80 mg/kg was better for a higher number of outcome measures. These data also point future studies to assess time-courses of mitochondrial dysfunction following mbTBI, as therapeutic windows vary by brain region and cellular source.

We have published on distinct differences in the response of synaptic versus glia-enriched mitochondria following experimental TBI.^52^ We find that rmbTBI produces synaptic bioenergetic changes, but not glia-enriched bioenergetic changes. This highlights the strength of the mitochondrial fractionation approach, as the synaptic effects could be hidden if examining total mitochondria from all cell types. Indeed, synaptic alterations have been described following mbTBI.^70,71^ Konan and co-workers reported that there are ultrastructural abnormalities in mitochondrial appearance, which coincide with synaptic deficits following mbTBI.^18^ These results support our findings of robust dysfunction in synaptic mitochondrial bioenergetics after rmbTBI.

The disconnection between mitochondrial impairment and oxidative damage in glia-enriched and synaptic mitochondria is a key finding of this study, which challenges dogma that these two processes go hand in hand. Indeed, recent findings by our group also showed that synaptic bioenergetic changes do not correspond to alterations in oxidative stress markers following a controlled cortical impact TBI model.^51^ An important point is that cytosolic levels of oxidative stress were not measured in this study, which could give an overall picture of cellular oxidative damage. Further, it is possible that calcium overload is contributing to synaptic mitochondrial dysfunction after mbTBI, rather than oxidative damage.^72^

Although rmbTBI does not result in overt bioenergetic changes in glia-enriched mitochondria, there may be other mitochondrial responses occurring in glia, such as astrocytes. Indeed, researchers have shown that there is a shift in the mitochondrial dynamics of astrocytes, namely elevation of mitochondrial fission, following *in vitro* and *in vivo* blast exposure.^73^ This could underlie the increases in oxidative stress that were observed in the current study.

The lack of changes in synaptic mitochondrial complex activity or synaptic mitochondrial-derived oxidative damage after rmbTBI points to potential impairments in key tricarboxylic acid (TCA) cycle intermediates or enzymes, such as pyruvate hydrogenase. Indeed, several reports show alterations in these enzymes after TBI, attributed to mitochondrial energy imbalance.^74,75^ One study demonstrates that energy crisis following bTBI may be due to deficits in mitochondrial glutamate oxaloacetate transaminase, also known as aspartate aminotransferase, which is a key enzyme responsible for conversion of TCA cycle intermediates.^14^ Although, the ability of MP201 to increase complex II and complex IV activity in synaptic mitochondria in the PFC could, in part, explain general MP201-induced increases in synaptic mitochondrial bioenergetics in the PFC.

Only male rats were used in this study and growing evidence suggests that sex plays a key role in outcomes following mbTBI.^12,76,77^ Future studies are needed on potential sex differences in mitochondrial outcomes following experimental TBI. Early pathological events, including mitochondrial impairment, contribute to ongoing neurological impairment after rmTBI. Future studies are planned to examine the effect of mild mitochondrial uncoupling therapy on subacute and chronic behavioral dysfunction.

This study highlights the overarching research that demonstrates the efficacy of a “mitoceutical” approach for treating TBI.^78^ Overall, these results provide seminal data on diffuse and robust alterations in synaptic mitochondrial bioenergetics following rmbTBI. Further, mild mitochondrial uncoupling can restore mitochondrial bioenergetics and oxidative balance and represents an effective strategy at targeting early metabolic crisis after mTBI.

## Transparency, Rigor, and Reproducibility Summary

The analysis plan was not formally pre-registered, but the team member with primary responsibility for the analysis certifies that the analysis plan was pre-specified.^1,2^ A sample size of eight subjects per group was planned based on ANOVA-based power analysis with the following parameters: α = 0.05, 1 − β = 0.8, and SD 20% of mean for experimental groups.^3^ Primary outcomes for sample size determination were State III mitochondrial bioenergetics.^3^ Forty-eight rats were subjected to experimental injury and randomly assigned to groups using a random number generator.^4,5^ Eight rats were assigned to each treatment group and complete data were obtained for six to eight/group as some assays resulted in technical issues.^4^ Investigator blinding was not fully possible because of the nature of the therapeutic intervention, but blinding was performed based on dose-response of the therapeutic.^6^

Therapeutic administration occurred 15 ± 2 min after injury and then daily. Dose-response relationship was performed using two doses, with both doses demonstrating efficacy in targeting outcome measures.^7^ The prodrug was received in a single batch from the company and fresh drug formulations were prepared weekly. The therapeutic was kept sealed, temperature controlled, and away from direct sunlight to ensure consistent activity.^8^ The normal distributions of primary respiration and blot intensity outcome data were verified using Shapiro-Wilk tests, and Brown-Forsythe and Bartlett's tests were performed to ensure homogeneity of variance.^9^ Correction for multiple comparisons was performed with Sidak's and Dunnett's post hoc tests.^10^ No replication or external validation studies have been performed or are planned/ongoing at this time to our knowledge.^11^ Data from this study are not available in a public archive. Data from this study will be made available (as allowable according to institutional regulatory standards) by e-mailing a corresponding author.^12^ The prodrug used to conduct the study is not publicly available.^13^ The authors agree or have agreed to publish the article using the Mary Ann Liebert, Inc. “Open Access” option under appropriate license.^15^ Portions of the Methods section found in this article were previously uploaded to a pre-print server; contact a corresponding author for the server access information.
